# Structure of thymidylate kinase from *Ehrlichia chaffeensis*

**DOI:** 10.1107/S174430911101493X

**Published:** 2011-08-16

**Authors:** David J. Leibly, Jan Abendroth, Cassie M. Bryan, Banumathi Sankaran, Angela Kelley, Lynn K. Barrett, Lance Stewart, Wesley C. Van Voorhis

**Affiliations:** a Seattle Structural Genomics Center for Infectious Disease (SSGCID), USA; b Department of Allergy and Infectious Diseases, School of Medicine, University of Washington, Box 356423, Seattle, WA 98195, USA; c Emerald BioStructures Inc., 7869 NE Day Road West, Bainbridge Island, WA 98110, USA; d Berkeley Center for Structural Biology, Lawrence Berkeley Laboratory, 1 Cyclotron Road, BLDG 6R2100, Berkeley, CA 94720, USA

**Keywords:** thymidine 5′-phosphate, thymidine 5′-diphosphate, *Ehrlichia chaffeensis*, human monocytotropic erlichiosis, thymidylate kinases

## Abstract

The enzyme thymidylate kinase phosphorylates the substrate thymidine 5′-­phosphate (dTMP) to form thymidine 5′-diphosphate (dTDP), which is further phosphorylated to dTTP for incorporation into DNA. *Ehrlichia chaffeensis* is the etiologic agent of human monocytotropic erlichiosis (HME), a potentially life-threatening tick-borne infection. HME is endemic in the United States from the southern states up to the eastern seaboard. HME is transmitted to humans *via* the lone star tick *Amblyomma americanum*. Here, the 2.15 Å resolution crystal structure of thymidylate kinase from *E. chaffeensis* in the apo form is presented.

## Abstract 2

A 2.15 Å resolution apo structure of thymidylate kinase from *E. chaffeensis* is reported.

## 1. Introduction

Thymidylate kinase (TMPK) phosphorylates the substrate thymidine 5′-phosphate (dTMP) to form thymidine 5′-diphosphate (dTDP). The overall reaction is as follows:
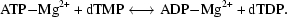
The newly formed dTDP is subsequently phosphorylated to dTTP by nucleoside-diphosphate kinase for incorporation into DNA. The essentiality of dTTP for DNA synthesis makes TMPK a desirable drug target (Kandeel *et al.*, 2009 ▶). There are ∼60 thymidylate kinase structures from 19 species currently deposited in the Protein Data Bank (PDB). The first of these protein structures was solved from herpes simplex virus type I (Wild *et al.*, 1997 ▶).

*Ehrlichia chaffeensis* is an obligate intracellular Gram-negative coccus bacterium. *E. chaffeensis* is the etiologic agent of a zoonotic infection occurring in a deer–tick cycle and is spread *via* the lone star tick *Amblyomma americanum* to the white-tailed deer *Odocoileus virginianus* and occasionally to humans. The lone star tick is primarily found in the southern and southeastern United States. *E. chaffeensis* is the causative agent of human monocytotropic ehrlichiosis (HME).

HME was first identified in 1987. Between its discovery and 2005 there were a total of 2396 reported cases of HME, with 471 occurring in 2005 and a trend of increasing infections from 2001 to 2005. HME can present as a mild asymptomatic infection. The most common symptoms, found in over 50% of patients, include fever, headache, malaise, myalgia and nausea (Dumler *et al.*, 2007 ▶). Current treatment for HME consists of the antimicrobial doxycycline, or rifampicin when doxycycline cannot be used owing to adverse reactions. Like many infectious diseases, there is a desire to develop better targeted drugs to treat HME. The mission of the Seattle Center for Structural Genomics (SSGCID) is to provide a blueprint for a structure-guided drug-design efforts.

## 2. Methods

### 2.1. Protein expression and purification

The gene encoding thymidylate kinase was amplified *via* PCR in a 96-well format using genomic DNA as a template. We used the ligase-independent cloning (LIC) technique (Aslanidis & de Jong, 1990 ▶). The primers are designed with an additional LIC sequence at the 5′ ends that is complementary to the LIC sequence in the plasmid vector (Mehlin *et al.*, 2006 ▶; Choi *et al.*, 2011 ▶). Purified PCR products were again cloned *via* LIC into the AVA0421 expression vector (Quartley *et al.*, 2009 ▶), which provides a cleavable hexahistidine tag at the N-­terminus of the expressed protein with the sequence MAHHH­HHHMGTLEAQTQ′GPGS (Choi *et al.*, 2011 ▶). The recombinant plasmids were then transformed into *Escherichia coli* Rosetta Oxford strain [BL21*(DE3)-R3-pRARE2] cells for expression testing. The University of Washington Protein Production Group (UW-PPG) utilizes recombinant human rhinovirus 3C protease MBP fusion (His-­MBP-3C protease) to cleave the hexahistadine tag (Bryan *et al.*, 2011 ▶). When the tag is cleaved the short GPGS sequence is left on the N-terminus of the full-length thymidylate kinase recombinant protein. The gene was assigned the SSGCID clone name EhchA.01616.a and will further be referred to as EhchA.01616.a/TMPK.

The transformed cells were tested for expression of soluble protein in a high-throughput screen and were then moved on to large-scale expression (Choi *et al.*, 2011 ▶). Starter cultures of LB broth with appropriate antibiotics were grown for ∼18 h at 310 K. ZYP-5052 auto-induction medium was freshly prepared as per UW-PPG standard protocols (Choi *et al.*, 2011 ▶; Studier, 2005 ▶). The bottles were inoculated with all of the overnight culture. Inoculated bottles were then placed into a LEX bioreactor (Harbinger, Ontario, Canada). Cultures were grown for ∼24 h at 298 K; the temperature was then reduced to 288 K and the culture was grown for a further ∼60 h. To harvest, the culture was centrifuged at 4000*g* for 20 min at 277 K. Cell paste was flash-frozen in liquid nitrogen and stored at 193 K.

Frozen recombinant cells were resuspended in a lysis buffer con­sisting of 25 m*M* HEPES pH 7.0, 500 m*M* NaCl, 5% glycerol, 0.5% CHAPS, 30 m*M* imidazole, 10 m*M* MgCl_2_, 1 m*M* tris(2-carboxy­ethyl)phosphine (TCEP), 250 µg ml^−1^ 4-(2-aminoethyl)benzenesulfonyl fluoride hydrochloride (AEBSF) and 0.025% sodium azide. The cells were ruptured *via* sonication, which was followed by incubation with benzonase nuclease (Invitrogen, Carlsbad, California, USA). The crude lysate was centrifuged at 31 500*g* and 277 K for 75 min and the supernatant was loaded onto a Nickel HisTrap FF 5 ml column (GE Healthcare, Piscataway, New Jersey, USA) for immobilized metal-affinity chromatography (IMAC). The column was washed with 20 column volumes of wash buffer (25 m*M* HEPES pH 7.0, 500 m*M* NaCl, 5% glycerol, 30 m*M* imidazole, 1 m*M* TCEP and 0.025% sodium azide). The bound protein was eluted with seven column volumes of elution buffer (25 m*M* HEPES pH 7.0, 500 m*M* NaCl, 5% glycerol, 1 m*M* TCEP, 250 m*M* imidazole and 0.025% sodium azide). Protein precipitation was observed in the elution fractions. An additional centrifugation step at 4000*g* was implemented to remove any insoluble aggregates that had formed. 1 m*M* adenosine diphosphate (ADP) and 1 m*M* MgCl_2_ were added to the soluble protein in an attempt to prevent further aggregation.

Cleavage of the N-terminal His tag was accomplished by overnight 277 K dialysis with His-MBP-3C protease in buffer consisting of 25 m*M* HEPES pH 7.5, 500 m*M* NaCl, 5% glycerol, 1 m*M* TCEP, 0.025% sodium azide, 1 m*M* ADP and 1 m*M* MgCl_2_. The cleaved protein was recovered in both the flowthrough and wash fractions of a second Ni^2+^-affinity chromatography step that also removed the His-MBP-3C protease, uncleaved protein and cleaved His tag. This IMAC clarification step utilized the same buffers as the initial IMAC purification. After affinity-tag cleavage, a tag remnant GPGS was left on the N-terminus of the full-length EhchA.01616.a/TMPK. Centrifugation at 43 000*g* for 30 min was performed to remove any precipitated protein that had formed during the cleavage/dialysis step. The soluble cleaved protein was further polished using a HiLoad 26/60 Superdex 75 prep-grade column (GE Healthcare) equilibrated with 25 m*M* HEPES pH 7.0, 500 m*M* NaCl, 5% glycerol, 2 m*M* dithiothreitol (DTT), 0.025% sodium azide, 1 m*M* ADP and 1 m*M* MgCl_2_. SDS–PAGE analysis was used to determine which fractions to pool. The purified protein was concentrated to 24 mg ml^−1^ and stored at 193 K.

### 2.2. Crystallization

Thawed protein was used to set up four sparse-matrix screens, JCSG+ (Emerald BioStructures, Bainbridge Island, Washington, USA), Crystal Screen and Index HT (Hampton Research, Aliso Viejo, California, USA) and PACT (Molecular Dimensions, Newmarket, Suffolk, UK), following an extended Newman strategy (Newman *et al.*, 2005 ▶). 0.4 µl protein solution was then mixed with 0.4 µl well solution and equilibrated against a 100 µl reservoir using 96-well Compact Jr crystallization plates (Emerald BioSystems). Crystals suitable for diffraction studies were found in condition G8 from the PACT screen: 100 m*M* Bis-Tris propane pH 7.5, 200 m*M* sodium sulfate, 20% PEG 3350. The crystals were cryoprotected with an additional 25% ethylene glycol.

### 2.3. Data collection and structure determination

A diffraction data set was collected on 2 December 2009 on ALS beamline 5.0.1 at the Berkeley Center for Structural Biology in the context of the Collaborative Crystallography program using a 3 × 3 tiled ADSC Q315r detector. 150 images were collected with a ϕ-­slicing of 1° per image. The diffraction data were reduced in space group *P*2_1_2_1_2_1_ to 2.15 Å resolution with *XDS*/*XSCALE* (Kabsch, 2010 ▶; Table 1 ▶).

The packing density (Matthews, 1968 ▶) suggested four molecules per asymmetric unit, with a *V*_M_ of 2.24 Å^3^ Da^−1^ and 45% solvent content. A search of the PDB for sequence homology yielded thymidylate kinase from *Aquifex aeolicus* (PDB entry 2pbr; J. Jeyakanthan, S. P. Kanaujia, C. Vasuki Ranjani, K. Sekar, N. Nakagawa, A. Ebihara, S. Kuramitsu, A. Shinkai, Y. Shiro & S. Yokoyama, unpublished work) as the closest sequence homolog, with 45% sequence identity. Molecular replacement was performed with the *CCP*4 (Winn *et al.*, 2011 ▶) program *Phaser* (McCoy *et al.*, 2007 ▶) using data between 20 and 3.5 Å resolution. The initial search model was modified with the *CCP*4 program *CHAINSAW* (Stein, 2008 ▶) based on sequence alignment with 2pbr. However, a search with the modified monomer *A* from 2pbr was not successful. A further truncation of the C-terminal residues 137–197 yielded convincing solutions for four monomers. Phases were improved with the *CCP*4 program *Parrot* (Cowtan, 2010 ▶) including NCS averaging. The *CCP*4 program *Buccaneer* (Cowtan, 2006 ▶) was then used to extend the initial model; the improved phases from *Parrot* were included during this process. 658 residues were built in 12 separate chains. The *R*_work_ of 0.385 and *R*_free_ of 0.429 indicated a rather incomplete model. The model from *Buccaneer* was then used for model extension in *ARP*/*wARP* (Langer *et al.*, 2008 ▶), which built 633 residues in 18 chains with significantly improved *R* factors: *R*_work_ = 0.228 and *R*_free_ = 0.324. The model was then iteratively extended manually using *Coot* (Emsley *et al.*, 2010 ▶) followed by cycles of reciprocal-space refinement with the *CCP*4 program *REFMAC*5 (Murshudov *et al.*, 2011 ▶). The final model could be refined with one TLS group per chain to an *R*_work_ of 0.187 and an *R*_free_ of 0.232 with good stereochemistry (Table 2 ▶). The model was validated with the validation tools in *Coot* and *MolProbity* (Chen *et al.*, 2010 ▶). The final model extends from residue Pro−2 to Gln200 for chains *A* and *C* and from Pro−2 to Met201 for chains *B* and *D*. In each chain residues 135–150 are too disordered to be modeled and there is a varying amount of disorder in the four chains between residues 178 and 189. There are two sets of Ramachandran outliers in this structure: Arg93 and Phe94 from each chain are located in a loop between a β-strand and an α-helix. The electron density for these two residues is well defined. The second set is the peptide bond between Pro−2 and Gly−1, which are part of the purification tag. The four chains almost superimpose and show good electron density; however, this peptide bond lies in the allowed Ramachandran region for two chains and in the disallowed region for the other two chains. One sulfate molecule from the precipitant could be located in each chain and some ethylene glycol from the cryoprotectant could be placed.

## 3. Results and discussion

### 3.1. Overall EhchA.01616.a/TMPK structure

Full-length EhchA.01616.a/TMPK could be purified with crystallizable quality. The full-length protein with the affinity-tag remnant sequence GPGS at the N-terminus crystallized rather readily and a 2.15 Å resolution data set was collected on ALS beamline 5.0.1 without further optimization of crystallization conditions. Despite high sequence identity (45%) to PDB entry 2pbr, molecular replace­ment was not straightforward. A significant C-terminal truncation was required for the search model to yield a solution. In hindsight, this could be explained by a larger structural difference between EhchA.01616.a/TMPK and 2pbr at the C-terminus compared with the N-terminus. A significant peak in a native Patterson map (20% height of the origin peak) indicated a pseudo-translational symmetry, which tends to complicate molecular-replacement searches.

The model of EhchA.01616.a/TMPK consists of four monomers per asymmetric unit. Interface analysis with *PISA* (Krissinel & Henrick, 1997 ▶) supports the presence of two separate dimers (*AB* and *CD*). The buried surface area was ∼1025 Å^2^ per monomer compared with a surface area of ∼9000 Å^2^ per monomer and the free binding energy was estimated as Δ*G* ^int^ = −84 kJ mol^−1^. The largest crystal-packing interface has a buried surface area of ∼600 Å^2^ and can only be found once in the crystal lattice. Dimers are typically observed for thymidylate kinases and the dimers of EhchA.01616.a/TMPK have the same quaternary structure as other TMPKs deposited in the PDB. Hence, we are confident that the dimer seen twice in this structure is the native dimer of EhchA.01616.a/TMPK.

**Figure 1:**
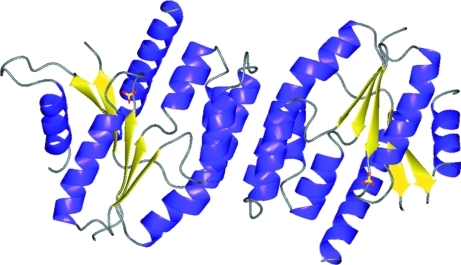
Dimer of EhchA.01616.a/TMPK formed by monomers *A* and *B*. The ribbons are colored by secondary structure. Two sulfate ions are shown as yellow/red sticks.

The fold seen for EhchA.01616.a/TMPK is as expected for TMPKs: a central five-stranded β-sheet is sandwiched between two α-helices on one side and five α-helices on the other  (Fig. 1 ▶). The four chains of EhchA.01616.a/TMPK are quite similar and superimpose with r.m.s.d.s of ∼0.4–0.5 Å for C^α^ atoms. An *SSM* search of the PDB for structural homologs reveals apo thymidylate kinase from *A. aeolicus* (PDB entry 2pbr) as the closest homolog, with r.m.s.d.s of around 1.1 Å and some distinct deviations of the C-termini. The second closest homolog is the structure of ligand-bound thymidylate kinase from *Thermotoga maritima* (PDB entry 3hjn; S. Yoshikawa, N. Nakagawa, M. Shirouzu, S. Yokoyama & S. Kuramitsu, unpublished work), with r.m.s.d.s in the range 1.3–1.4 Å.

**Figure 2:**
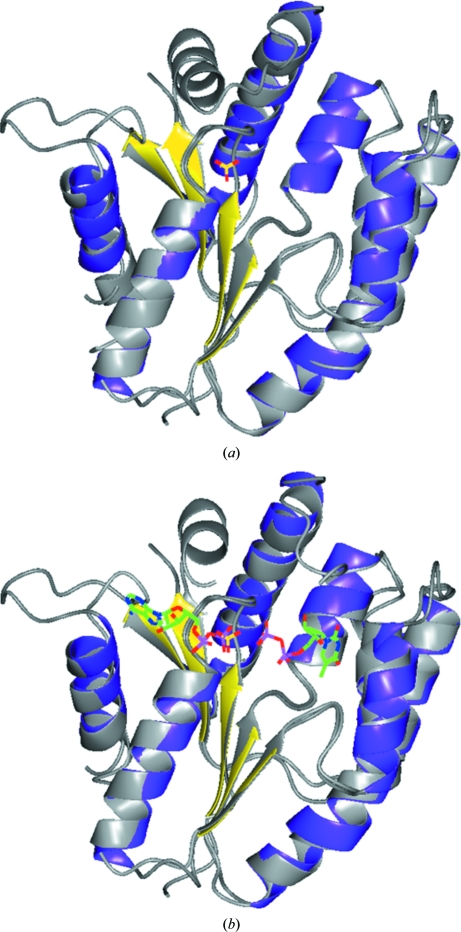
Superposition of EhchA.01616.a/TMPK monomer *A* with (*a*) thymidylate kinase from *A. aeolicus* (2pbr) and (*b*) thymidylate kinase from *T. maritima* (3hjn). In each figure the EhchA.01616.a structure is shown in the same colours as in Fig. 1 ▶, while the ribbons for TMPK from *A. aeolicus* and *T. maritima* are shown in light gray. Ligands for each structure are shown as coloured stick models. The sulfate ions in EhchA.01616.a/TMPK and *A. aeolicus* TMPK superimpose. They also superimpose with a phosphate of ADP in the *T. maritima* structure.

A sulfate ion could be located in each of the monomers of EhchA.01616.a/TMPK. We assume that the sulfate ion was provided by the crystallization buffer, which contained 200 m*M* sodium sulfate. The structure of TMPK from *A. aeolicus* shows a sulfate ion in the same location (Fig. 2 ▶ *a*). This protein was crystallized in the presence of 50 m*M* lithium sulfate. The structure of TMPK from *T. maritima* (PDB entry 3hjn) was crystallized in complex with adenosine 5′-­diphosphate (ADP) and thymidine 5′-diphosphate. The β-phosphate group of ADP in 3hjn superimposes with the sulfate in the other two structures. The nucleotide-binding pocket is structurally conserved between the ADP-bound *T. maritima* structure and the apo *E. chaffeensis* structure. Nucleotide binding would only require subtle structural changes that mostly involve side chains. As the binding pocket is accessible and is not blocked by the crystal lattice, it is likely that EhchA.01616.a/TMPK crystals will be soakable with nucleotides.

### 3.2. Comparison to human TMPK

EhchA.01616.a/TMPK has only 25% amino-acid sequence identity to the human TMPK protein. When compared with human TMPK bound to ADP, TMP and Mg^2+^ (PDB entry 1e2f; Ostermann *et al.*, 2000 ▶) there are a few observed structural differences. Most notable are the structural differences near the C-terminus. There is a loop found in the EhchA.01616.a/TMPK structure that is not observed in the human protein or PDB entries 2pbr or 3hjn. This loop appears to be a result of a five-amino-acid insertion from Tyr189 to Asp193. In the apo structure this loop is in close proximity to the ATP-binding site, with the loop oriented away from the binding site. It is unknown whether there are any conformational changes of the loop on nucleotide binding for the EhchA.01616.a/TMPK protein. It is also unknown whether this loop has any biological significance or whether this unique structural feature can be exploited for targeted drug development.

The P-loop nucleoside-binding motif (*GX*_4_GKS/T) found in many nucleotide-binding proteins is present in both the human and *Ehrlichia* TMPKs (Saraste *et al.*, 1994 ▶). Specifically, the P-loop amino-acid sequences of the human and *Ehrlichia* proteins are GVDRAGKS and GIDGSGKT, respectively. These motifs both contain an acidic Asp residue that is uniquely found in TMPKs compared with other nucleoside monophosphate kinases (Lavie *et al.*, 1998 ▶). The human enzyme is a type I TMPK, in which the Asp15 residue is immediately followed by a catalytically important arginine residue. The *Ehrlichia* protein is instead a type II TMPK, with the Asp9 residue being followed by a glycine residue (Lavie *et al.*, 1998 ▶). The P loops of the human and *Ehrlichia* enzymes have no major structural differences. The P loop is one of three regions known to undergo conformational changes on substrate binding (Ostermann *et al.*, 2000 ▶). Substrate-bound structures of EhchA.01616.a/TMPK would be needed in order to understand the conformational changes of the P-loop in comparison to those of the human protein. Given the difference in the catalytic importance of the P loop between type I and type II TMPKs, it may be possible to exploit this difference for drug design.

The flexible LID region also undergoes conformational changes and has catalytic differences between type I and type II TMPKs; the LID region closes upon ATP binding (Ostermann *et al.*, 2000 ▶). The LID region remains unmodeled in the apo EhchA.01616.a/TMPK structure. As for the P loop, substrate-bound structures would be needed to fully compare the EhchA.01616.a/TMPK and human TMPK LID regions. There is no evidence that the overall structure of the LID region of EhchA.01616.a/TMPK would be significantly different from that of the human protein. However, there are significant amino-acid differences between *Ehrlichia*, human and other type II TMPKs. The catalytic arginine found in the P loop of type I TMPKs is found in the LID region of type II TMPKs. Typically, type II TMPKs have several basic residues in the LID region; for example, *E. coli* TMPK contains five basic residues in the region as opposed to three in the human protein (Lavie *et al.*, 1998 ▶). The basic residues of the *E. coli* protein consist of Lys148, Arg149, Arg151, Arg153 and Arg158, with Arg153 assuming the catalytic role of Arg16 in the P loop of the human TMPK. The *Ehrlichia* protein only contains two basic residues in the LID region, Arg141 and Lys144, with Arg141 presumed to be the catalytic residue. Since we do not currently have substrate-bound EhchA.01616.a/TMPK structures to fully compare with the human protein, it is difficult to determine the ability to target the protein with a novel drug based on structural differences alone. Based on both the catalytic differences of the P loop and LID region and amino-acid sequence differences, there is a possibility of specifically targeting EhchA.01616.a/TMPK over the human homologue.

## 4. Conclusion

This paper describes a purification strategy that results in EhchA.01616.a/TMPK of crystallizable quality. The resulting 2.15 Å resolution crystal structure contained two dimers. While the fold is conserved within the TMPK family, significant changes are seen at the C-terminus which also have an impact on the molecular-replacement strategy. It is unknown whether there are biological implications of the difference in the C-terminus compared with other TMPKs. A sulfate ion from the crystallant occupies the β-phosphate position of the ADP observed in homologous structures. Furthermore, substrate-bound structures of EhchA.01616.a/TMPK would be beneficial to fully analyze the structural differences between the *Ehrlichia* and human proteins. At the time of publication, only nine structures of proteins from *E. chaffeensis* have been deposited in the PDB.

## Supplementary Material

PDB reference: thymidylate kinase, 3ld9

## Figures and Tables

**Table 1:** Data-collection statistics Values in parentheses are for the highest of 20 resolution shells.

Wavelength (Å)	0.9774
Space group	*P*2_1_2_1_2_1_
Unit-cell parameters (Å)	*a* = 39.17, *b* = 144.82, *c* = 146.84
Resolution range (Å)	50–2.15 (2.21–2.15)
Unique reflections	46708 (3410)
Multiplicity	5.9 (5.8)
Completeness (%)	100 (99.3)
*R*_merge_†	0.086 (0.529)
Mean *I*/σ(*I*)	16.8 (3.7)

† *R*_merge_ = 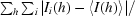 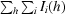.

**Table 2:** Refinement and model statistics Values in parentheses are for the highest of 20 resolution shells.

Resolution range (Å)	50–2.15 (2.21–2.15)
*R*_cryst_†	0.189 (0.205)
*R*_free_†	0.232 (0.268)
R.m.s.d. bonds (Å)	0.014
R.m.s.d. angles (°)	1.34
Protein atoms	5575
Nonprotein atoms	329
Wilson *B* factor (Å^2^)	26.1
Mean *B* factor (Å^2^)	31.5
Residues in favored region	656 [94%]
Residues in allowed region	22 [3.2%]
Residues in disallowed region	10 [1.5%]
*MolProbity* score [percentile]	1.61 [96th]
PDB code	3ld9

† *R*_cryst_ = 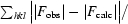 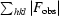. The free *R* factor was calculated using an equivalent equation with the 5% of the reflections that were omitted from the refinement.
